# Case report: Clinical and virological characteristics of aseptic meningitis caused by a recombinant echovirus 18 in an immunocompetent adult

**DOI:** 10.3389/fmed.2022.1094347

**Published:** 2023-01-12

**Authors:** Chunmei Jiang, Zhixiang Xu, Jin Li, Jiaqi Zhang, Xingkui Xue, Jingxia Jiang, Guihua Jiang, Xisheng Wang, Yun Peng, Tian Chen, Zhenzhen Liu, Liu Xie, Haibin Gao, Yingxia Liu, Yang Yang

**Affiliations:** ^1^Department of Infectious Disease, The People’s Hospital of Longhua, Shenzhen, China; ^2^Shenzhen Key Laboratory of Pathogen and Immunity, State Key Discipline of Infectious Disease, Shenzhen Third People’s Hospital, Second Hospital Affiliated to Southern University of Science and Technology, Shenzhen, China; ^3^Shenzhen Polytechnic, Shenzhen, China

**Keywords:** aseptic meningitis, echovirus 18, immunocompetent adult, recombination < evolution, case report

## Abstract

Echovirus 18 has been recognized as an important causative pathogen of aseptic meningitis in young children worldwide, and echovirus 18-induced meningitis is rarely found in adults with immunocompetence. In this case study, we report the clinical and virological characteristics of aseptic meningitis caused by recombinant echovirus 18 in an adult with immunocompetence. A 31-year-old woman with immunocompetence was admitted to our hospital with fever, dizziness, severe headache, nausea, and vomiting for the past 1 day and was diagnosed with viral meningitis based on the clinical manifestations and laboratory results from cerebrospinal fluid (CSF). The patient received antiviral treatment with ribavirin and interferon as soon as the enterovirus infection was identified using qRT-PCR and was cured after 4 days. From the oropharyngeal swab and CSF samples, two echovirus 18 strains were isolated with a single nucleotide difference located at the 5′ UTR. Phylogenetic analyses based on the VP1 gene showed that the two strains belonged to the subgenotype C2 and were clustered with sequences obtained from China after 2015, while the results from the 3D polymerase region showed that the two strains were closely related to the E30 strains. Bootscanning results using the 5′ UTR to 2A region and the 2B to 3′ UTR region showed that potential intertypic recombination had occurred in the 2B gene. Recombination analyses further confirmed that the two strains (echovirus 18) presented genome recombination with echovirus 30 in the nucleotide regions of the 2B gene. To the best of our knowledge, this is the first report of echovirus 18-induced meningitis in an adult with immunocompetence from mainland China, highlighting the need for close surveillance of echovirus 18 both in children and adults in the future.

## Introduction

Meningitis is inflammation of the meninges characterized by an abnormal number of white blood cells (WBC) in cerebrospinal fluid (CSF) with few or no focal neurological findings or brain abnormalities on imaging and accompanied by the acute onset of fever, headache, and neck stiffness (1, 2). Infectious agents, including viruses, bacteria, and fungi, are the most common causes of meningitis (3). Viral meningitis is associated with the acute onset of meningeal symptoms and fever, pleocytosis of the cerebrospinal fluid, and no growth on routine bacterial cultures, usually affecting young children (1, 4). Many viruses have been shown to cause viral meningitis, such as enteroviruses (EVs), parechovirus, herpesviruses, influenza viruses, arboviruses, and coronaviruses, and viral etiology varies according to age and country (3).

Human enteroviruses belong to the Enterovirus genus of the Picornaviridae family and are characterized as non-enveloped, positive-sense, single-stranded RNA viruses with ∼7,500 nucleotides (5). The enteroviruses contain four enterovirus species (A to D) and three rhinovirus species (A to C) and infect millions of people worldwide every year with mostly mild and self-limited symptoms, such as hand-foot-and-mouth disease, herpangina, pleurodynia, rashes, and rhinitis (5, 6). Moreover, an increasing number of enteroviruses have been found to infect the central nervous system and result in various neurological diseases, of which non-polio human enteroviruses (NPEV) have become the leading recognizable cause of viral meningitis (5, 6). Notably, Enterovirus B (EV-B), a species of the genus Enterovirus, particularly echovirus 6, 9, 13, 14, 16, and 30, accounted for the majority of meningitis cases with worldwide distribution (5). As a member of EV-B, echovirus 18 was first discovered in 1955 (7), and since then, it has been reported as a pathogen causing aseptic meningitis in many countries of the world (8–14). Most of the patients in these reports were young children, and an outbreak of encephalitis/meningitis caused by echovirus 18 in children has been reported in 2015 in mainland China (9). There was no report of echovirus 18-induced meningitis in adults with immunocompetence in mainland China and scant report in other countries of world (10–12). In the present study, we report the clinical and virological characteristics of aseptic meningitis caused by the recombinant echovirus 18 in an adult with immunocompetence from Shenzhen, China.

## Materials and methods

### Sample collection and virus isolation

Oropharyngeal swabs and CSF samples were collected from the patient on the first and second days following admission, respectively. All specimens positive for enterovirus infection by qRT-PCR were cultured in Rhabdomyosarcoma (RD) cells for virus isolation. The study was performed in accordance with guidelines approved by the ethics committees of Shenzhen People’s Hospital Longhua Branch and the Shenzhen Third People’s Hospital. Written informed consent was obtained from the patient for the publication of any potentially identifiable images or data included in this article.

### Quantitative reverse transcriptase PCR (qRT-PCR)

Nucleic acids were extracted from 140 μl of the oropharyngeal swab, CSF, and cell culture supernatant samples using the QIAamp RNA Viral Kit (Qiagen, Germany) according to the instructions of the manufacturer. Nucleic acids were eluted in 50 μl of AVE buffer and stored at −80°C for subsequent use. Nucleic acids samples were tested by quantitative reverse transcription polymerase chain reaction (qRT-PCR) in an ABI QuantStudio Dx Real-Time cycler (Applied Biosystems, USA), using commercial kits targeting the common pathogens that induce meningitis (Mabsky Biotech Co., Ltd.), including herpes simplex virus (HSV)-1, HSV-2, varicella-zoster virus (VZV), human cytomegalovirus (HCMV), Epstein-Barr virus (EBV), human herpes virus 6 (HHV-6), HHV7, JC virus (JCV), human parechovirus (HPeV), enterovirus (EV), mumps virus, measles virus, *Haemophilus influenzae*, *Streptococcus pneumoniae*, *Staphylococcus aureus*, *Listeria monocytogenes*, *Neisseria meningitidis*, *Streptococcus agalactiae*, *Cryptococcus neoformans*, and *Escherichia coli*.

### Full-length genome amplification

The amplification and sequencing of our two echovirus strains were performed, as previously reported with some modifications (15). Three long-distance PCR amplifications were performed using a PrimeScriptTM One-Step RT-PCR Kit Version 2 (Takara, China). The primers used for RT-PCR and sequencing of the full-length genome were designed using a “primer-walking” strategy and are listed in Supplementary Table 1. The PCR products were purified using the QIAquick PCR purification kit (Qiagen, Germany) and sequenced (Sangon, China).

### Phylogenetic analysis

The phylogenetic analyses were done as previously reported with slight modifications (16). Sequence alignment was performed using MEGA software version 7.0. Phylogenetic trees of the 5′ UTR to 2A region, the 2B to 3′ UTR regions, and the 3D gene were constructed by the neighbor-joining method, with bootstrap values obtained from 1,000 replicates and bootstrap values of >80% were shown. The phylogenetic tree of the VP1 gene and the evolution rates were constructed and determined with Bayesian Evolutionary Analysis Sampling Trees (BEAST) software version 1.8 using a lognormal relaxed clock, a constant-size tree prior, and the GTR + G substitution model. Each Bayesian MCMC analysis was run for 100 million generations. Bootstrap testing with 1,000 replicates was used to estimate the strength of the phylogenetic trees.

### Recombination analysis

The recombination analyses were done as described in the previous report (16). A potential recombination within the complete genome sequences of echovirus 18 was assessed using the similarity plot and the bootscanning method with the neighbor-joining method and the Kimura 2-parameter substitution model with a window size of 500 nucleotides (nt) and a step size of 20 nt. The value of the permuted trees of > 80% indicated potential recombination events.

## Results

### Case presentation

**FIGURE 1:**
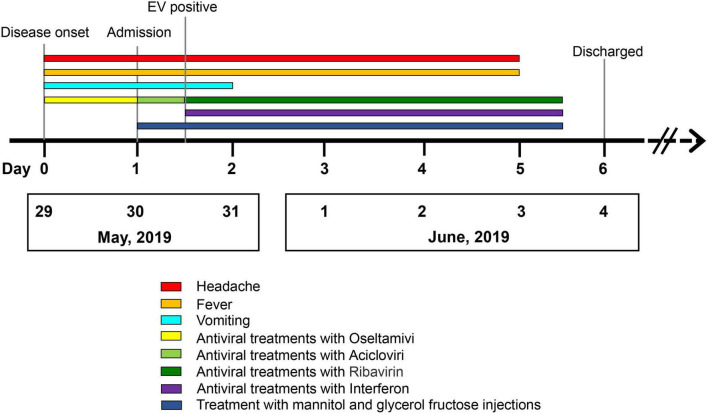
The timeline of the patients with echovirus 18-induced aseptic meningitis.

A 31-year-old immunocompetent woman was admitted to our hospital in May 2019 and presented with fever (37.8°C), dizziness, severe headache, nausea, and vomiting for 1 day (Figure 1). Upon admission, the patient had a normal heart rate (71 bpm) and blood pressure (116/76 mmHg). There were no abnormalities in the skin, in muscle tension, and during defecation, and there was no manifestation of Babinski’s sign but of nuchal rigidity. She was primarily diagnosed as having an intracranial infection based on the clinical manifestations above and hospitalized (4). Serum laboratory tests revealed leukopenia (WBC count: 3.28 × 10^9^/L) and lymphopenia (LYM count: 0.97 × 10^9^/L). Furthermore, pleocytosis (WBC in CSF: 40 × 10^6^/L) (17, 18), moderately increased levels of protein (564 mg/dl), and normal glucose (2.93 mmol/L) were found in CSF (4) (Table 1). Based on the manifestations and laboratory results, viral meningitis was diagnosed. To reduce the intracranial pressure, an empirical therapy with intravenous acyclovir for antiviral treatments and mannitol and glycerol fructose injections was used (Figure 1). A panel of common pathogens that induce meningitis was detected using commercial qRT-PCR kits, and the results showed that EV was positive in both the oropharyngeal swab and the CSF samples. Then, ribavirin and interferon were used as soon as possible instead of acyclovir. The fever lasted for another 3 days with a higher body temperature of 38.4°C, and the headache also resolved completely 3 days after the therapy. The treatment with interferon, mannitol, and glycerol fructose injections was stopped after 12 h of the fever breaks. Meanwhile, the result of the Gram staining smear test was negative 1 day after submission, and the bacterial culture with CSF was also negative during the cultivation time, which further confirmed aseptic meningitis.

**TABLE 1:** Laboratory results of the serum and cerebrospinal fluid.

Parameter	Sample type	Laboratory results	Reference range	Interpretation of result
WBC (× 10^9^/L)	Serum	3.28	3.5–9.5	Decreased
LYM (× 10^9^/L)	Serum	0.97	1.1–3.2	Decreased
LYM (%)	Serum	29.6	20–50	Normal
NEU (× 10^9^/L)	Serum	1.78	1.8–6.3	Decreased
NEU (%)	Serum	54.3	40–75	Normal
MONO (× 10^9^/L)	Serum	0.51	0.1–0.6	Normal
MONO (%)	Serum	15.5	3–10	Increased
EO (× 10^9^/L)	Serum	0.01	0.02–0.52	Decreased
EO (%)	Serum	0.3	0.4–0.8	Decreased
BASO (× 10^9^/L)	Serum	0.01	0.00–0.06	Normal
BASO (%)	Serum	0.3	0.1–1	Normal
PLT (× 10^9^/L)	Serum	227	125–350	Normal
WBC (× 10^6^/L)	CSF	40	0–8	Increased
RBC (× 10^6^/L)	CSF	0	0	Normal
GLU (mmol/L)	CSF	2.93	2.8–4.5	Normal
CI (mmol/L)	CSF	125.9	120–130	Normal
PRO (mg/dL)	CSF	564	0–500	Increased

WBC, white blood cell; LYM, lymphocyte; NEU, neutrophil; MONO, monocyte; EO, eosinophils; BASO, basophilic granulocyte; PLT, platelet; RBC, red blood cell; GLU, glucose; CI, chloridion; PRO, protein.

### Isolation and identification of echovirus 18

The product of qRT-PCR was sequenced using Sanger sequencing, and the results of BLAST showed the highest similarity with echovirus 18. Then, the oropharyngeal swab and CSF samples were subjected to virus isolation using RD cells. After two rounds of passage, both the supernatant from the oropharyngeal swab and the CSF samples showed typical cytopathic effects (CPEs) and positive qRT-PCR results of EV, indicating the successful isolation of the virus (data not shown). Overlapping fragments covering the entire genome of the virus were amplified and then sequenced by Sanger sequencing. The assembled genome sequences were submitted to the GenBank database with strain names LJ/0530/2019 (GenBank accession no. MN215884) and LJ/0601/2019 (GenBank accession no. MN337405) from oropharyngeal swab and CSF samples, respectively. Only one nucleotide at the 5′ UTR differed between the two strains with C in the CSF and T in the oropharyngeal swab, and this difference was confirmed using the original specimens. The complete genomes of the two strains showed an identity of 89.07% with the prototype strain of echovirus 18 strain Metcalf (GenBank accession no. AF317694). Meanwhile, the two strains showed the highest similarity of approximately 99% with the two sequences collected in Beijing, China, in 2018 (GenBank accession no. MN815810.1 and MN815811.1).

### Phylogenetic characteristics of the isolated echovirus 18

**FIGURE 2:**
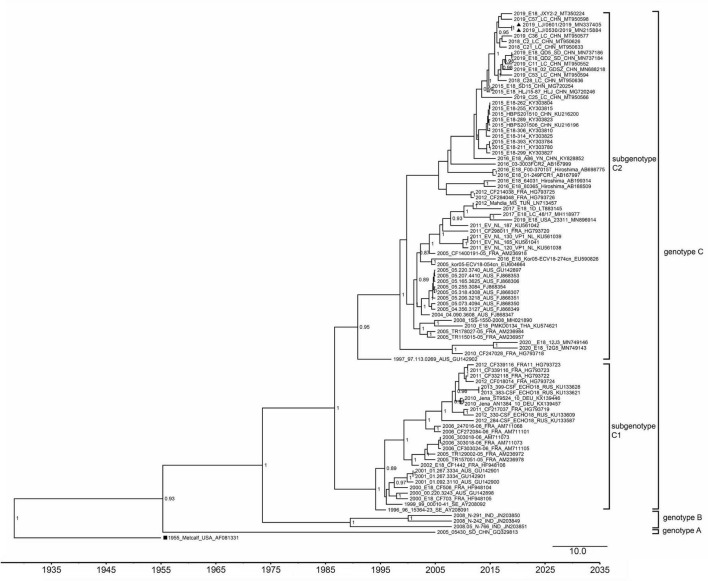
Phylogenetic tree based on the VP1 gene of echovirus 18. The phylogenetic tree was constructed using BEAST software. The posterior probabilities from the MrBayes analyses supporting the trees are indicated at the nodes. The triangle indicates the two strains in our study. The scale bar indicates years. The strain name, year of sampling, and GenBank accession numbers are shown.

To analyze the evolutionary relationship of the two strains to the other 18 echovirus strains in our study, phylogenetic analyses based on the VP1 coding region and 3D polymerase genes were performed. A total of 95 representative complete VP1 gene sequences of echovirus 18, including the 14 strains dated in 2019 and 2020, were downloaded from GenBank and analyzed using MEGA7.0 and BEAST software. As previously reported (9), all the strains were divided into three genotypes (genotypes A, B, and C), and genotype C was further divided into the C1 and C2 subgroups (Figure 2). The mean genetic distance between genotypes A and B was 0.17 (ranging from 0.15 to 0.21), between genotypes A and C was 0.22 (ranging from 0.19 to 0.25), and between genotypes B and C was 0.19 (ranging from 0.15 to 0.24). The two strains in our study belonged to the subgenotype C2 and were clustered with sequences obtained in China after 2015. Phylogenetic analysis of 3D the polymerase region was conducted using sequences obtained from the 3D gene of the LJ-0601 and its first 100 hits obtained from a BLAST search in GenBank (Supplementary Figure 1). Our two strains were also found to be closely related to the E30 strain (GenBank accession no. MW080372, MW080377, and MK238483), which differs from the results with the VP1 gene, indicating that possible recombination has occurred. Notably, strains E18-221, E18-398, and E18-314 were also found to be recombinants based on our analysis, which is consistent with a previous report (16).

**FIGURE 3:**
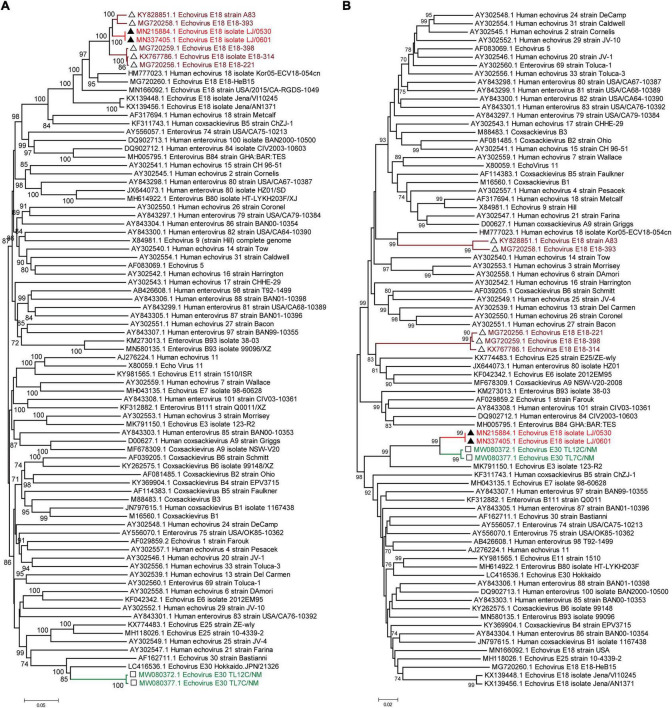
Phylogenetic relationships based on the 5′ UTR to 2A region and the 2B region to 3′ UTR of EV-B strains. Panels **(A,B)** show the phylogenetic trees of the representative 5′ UTR to 2A region **(A)** and the 2B to 3′ UTR region of the EV-B strains. Numbers at the nodes indicate bootstrap support for that node (percentage of 1,000 bootstrap replicates). The scale bars represent the genetic distance. Red indicates the two strains in our study. Brown indicates the echovirus 18 strains that are highly homologous to our strains in the 5′ UTR to 2A region. Green indicates the echovirus 30 strains that are highly homologous to our strains in the 2B region to 3′ UTR.

Moreover, phylogenetic relationship analyses based on the bootscanning results were performed using the 5′ UTR to 2A region (Figure 3A) and the 2B to 3′ UTR region (Figure 3B) of our two strains and the other 79 EV-B strains available in GenBank. The results showed that the 5′ UTR to 2A region of our two strains was closely clustered with other echovirus 18 strains, especially the strains found in Hebei, China, in 2015 (Figure 3), which is consistent with the preliminary molecular typing results. However, for the 2B to 3′ UTR region, our two strains did not cluster with other echovirus 18 strains while showing high similarity with the two echovirus E30 strains (GenBank accession no. MW080372 and MW080377), as shown in Supplementary Figure 1, suggesting that potential intertypic recombination had occurred in the 2B gene.

### Recombination analysis of the isolated echovirus 18

**FIGURE 4:**
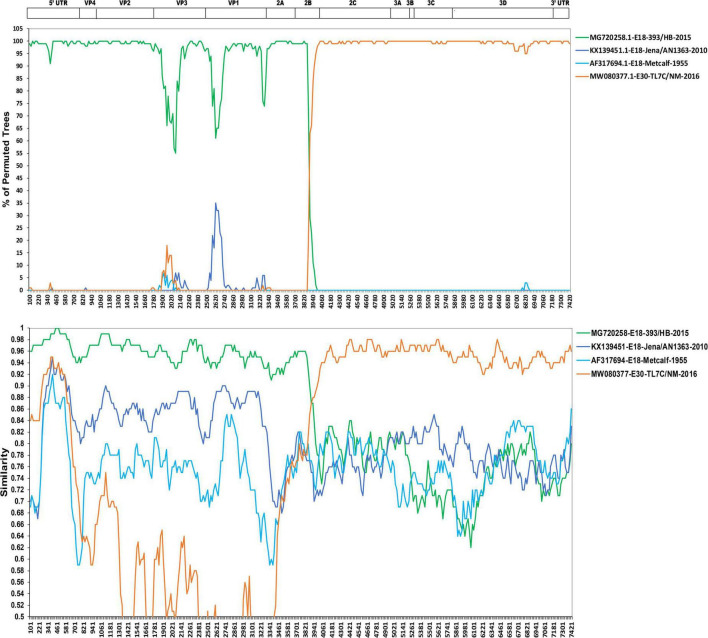
Similarity plot and bootscanning analyses of the two echovirus 18 strains based on their full-length genomes. Four potential parental strains of echovirus 18 and echovirus 30 were subjected to similarity plots and bootscanning analyses using Simplot v3.1 software.

First, the RDP V4.1 software was used to analyze the potential recombinant sequences (19). The results showed that the most possible major and minor parent strains were echovirus 18 isolate E18-393/HeB/CHN/2015 (GenBank accession no. MG720258) and echovirus E30 isolate TL7C/NM/CHN/2016 (GenBank accession no. MW080377), respectively, and other possible parent strains include echovirus 18 isolate Metcalf (GenBank accession no. AF317694) and echovirus E30 isolate TL7C/NM/CHN/2016 (GenBank accession no. MW080377) (data not shown). By combining the results of the phylogenetic and RDP analyses, four potential parental strains were subjected to similarity plots and bootscanning analyses using Simplot v3.1 software (Figure 4). Consistent with the results in Figure 3, our two strains showed the highest similarity with the echovirus 18 isolate E18-393/HeB/CHN/2015 in the 5′ UTR to 2A region and with the echovirus E30 isolate TL7C/NM/CHN/2016 in the 2B to 3′ UTR region. Furthermore, the bootscanning results further confirmed that the intratypic recombination site was in the 2B gene (Figure 4).

## Discussion

For the clinical diagnosis of meningitis, clinical symptoms are always not typical enough, as fever, chills, abdominal pain, nausea, and headaches are common in patients with meningitis, and short and fast breathing, loss of appetite, neck stiffness and pain, and sensitivity to bright light were also found in some patients (2, 20). Meanwhile, the presence of Brudzinski’s sign, Kering’s sign, or nuchal rigidity indicates that these bedside tests can be used by physicians to assess whether a patient should have a lumbar puncture (20). WBC, the proportion of neutrophils, glucose, and concentration of lactic acid in the CSF are key for differentiating the different types of meningitis (20–23). Although bacterial meningitis is usually characterized as a high WBC count (≥ 500 cells/μl) with a large proportion of neutrophils (>80%), studies also found that patients with bacterial meningitis may present with normal CSF leukocyte counts (24, 25), which is of concern for the diagnosis based on a lumbar puncture. Moreover, with few exceptions, the clinical manifestations and symptoms associated with viral meningitis are similar despite different causative viruses, making it difficult to choose antiviral agents (1, 4). Currently, molecular diagnosis methods, including nested-PCR, multiplex PCR, and qRT-PCR assays targeting specific viruses, serve as the new golden standard tool for the diagnosis of viral meningitis with high sensitivity and specificity, which enables healthcare providers to rapidly diagnose certain infections and therefore allow clinical management decisions (4, 26). In our study, the patient received empirical antiviral therapy with intravenous acyclovir upon admission, which is effective for Herpesviridae family members but not for enteroviruses (27). The antiviral agents were changed into ribavirin and interferon as soon as the EV infection was identified using qRT-PCR, which could significantly improve the antiviral treatment efficiency with the rapid cure of this patient.

Aseptic meningitis encompasses broad differential diagnoses related to inflammation of the meninges not caused by pyogenic bacteria with both infective and noninfective causes, while viral pathogens are the most common causative agents (20). Notably, the viral etiology varies by age, the immune status of the host, geographic location, season, and exposure history (2). For example, arbovirus-induced meningitis was highly correlated with geographical location and season; Herpesviridae family members, including CMV, VZV, HHV-6, and EBV-induced meningitis, were mainly found in patients who were immunocompromised but rarely in individuals with immunocompetence (28); mumps, measles, and VZV-induced CNS infections significantly declined after effective vaccines became available (2). Enteroviruses are the most common viruses that could cause meningitis in children and adults (5, 29). Studies showed that approximately 58.6% of the infected children and 51.6% of the infected adults diagnosed with meningitis/encephalitis were due to enterovirus (30, 31). One recent nationwide active surveillance study in China showed that EVs accounted for approximately 64.14% of acute meningitis/encephalitis in children and 25.19% in adults (32).

Echovirus 18-induced aseptic meningitis was mainly found in children, from sporadic infection to outbreak in various countries (12, 14, 15, 33–36), and the most recent outbreak in children has been reported in 2015 in mainland China (9). Adult patients with echovirus 18-induced aseptic meningitis have been previously reported in a limited number of cases (10, 12, 14), while severe manifestations and possibly long-term neurofunctional disability were also found to be associated (37, 38). Studies showed that the echovirus 18 strains were phylogenetically divided into three genotypes (16), and our strain belonged to the C1 subgroup, clustered with strains from China found after 2015. Moreover, the results based on the bootscanning analyses using the 5′ UTR to 2A region and the 2B to 3′ UTR region suggested a potential intratypic recombination had occurred in the 2B gene, and this was further confirmed by the recombination analysis. Recombination and mutations in enteroviruses have been recognized as the main mechanisms for the observed high rate of evolution and subsequently lead to changes in the tropism and virulence of enterovirus (39, 40). Similar to other enteroviruses, recombination was also found to be frequent for echovirus 18 (15, 16). Previous studies suggested that the current echovirus 18 strains were potential multiple-recombinant viruses containing many other EV-B donor sequences, and recombination was frequently detected in the 5′ UTR, P2, and P3 regions (15, 16). Consistent with previous studies, the recombination site of our strain was also in the 2B protein of the P2 region. Moreover, as studies showed that mutations and deletions in the UTR of some viruses are associated with replication and virulence (41–45), the single nucleotide difference between the two strains from the CSF and oropharyngeal swab samples in the 5′ UTR of echovirus 18 merits further investigation.

## Conclusion

Although echovirus 18 has been circulating in mainland China for years, induced aseptic meningitis by echovirus 18 was only found in children previously. To the best of our knowledge, this is the first report of echovirus 18 in an adult with immunocompetence from mainland China, highlighting the need for close surveillance of echovirus 18 both in children and adults in the future.

## Data availability statement

The datasets presented in this study can be found in online repositories. The names of the repository/repositories and accession number (s) can be found in the Genbank database: MN215884 and MN337405.

## Ethics statement

This study was performed in accordance with guidelines approved by the Ethics Committees from the People’s Hospital of Longhua and Shenzhen Third People’s Hospital. Written informed consent was obtained from the patient for the publication of any potentially identifiable images or data included in this article.

## Author contributions

YY, YL, and HG contributed to the conception and design of the study. CJ, ZX, JL, JZ, and XX enrolled the patient, performed the experiments, and analyzed the clinical data and sequences. JJ, GJ, XW, YP, TC, ZL, and LX collected the clinical data and performed some of the experiments. JL and YY drafted the manuscript. CJ, HG, and YL contributed to the critical revision of the manuscript. All authors reviewed and revised the manuscript and approved the final version.
