# Supplementary material for: Case report: Clinical and virological characteristics of aseptic meningitis caused by a recombinant echovirus 18 in an immunocompetent adult
Source: Front Med (Lausanne). 2023 Jan 12;9:1094347. doi: 10.3389/fmed.2022.1094347 (PMC9878155; doi:10.3389/fmed.2022.1094347)
Supplement: Supplementary file 1 [file Data_Sheet_1.docx]

**Supplementary materials**

**Figure S1.** Phylogenetic relationships based on the 3D polymerase region.

**

**

**Table S1. Primers used for complete genome amplification and sequencing.**

| Primers | Sequences (5’-3’) | Nucleotide position* |
| --- | --- | --- |
| Echo-18-1F | TATAACAGCCTGTGGGTTG | 2-20 |
| Echo-18-1R | GTAAGTCCCGTCCTGTGT | 3039-3056 |
| Echo-18-2F | ATGTCAATTCCATTCATTAGCGT | 2973-2995 |
| Echo-18-2R | CACANACTARCGGGCAACA | 4896-4914 |
| Echo-18-3F | ACATGCCRATGTCAGTGAAG | 4837-4856 |
| Echo-18-3R | ACCGAATGCGGAGAATTTAC | 7391-7410 |

*Numbering according to the genome of echovirus 18 strain E18-314/HB/CHN/2015.
